# Epidemiology of Aging in at risk populations

**DOI:** 10.1093/geroni/igaf122.511

**Published:** 2025-12-31

## Abstract

In this session you will gain insights into rural health as well as substance use disorders

